# Cell DiffErential Expression by Pooling (CellDEEP) highlights issues in differential gene expression in scRNA-seq

**DOI:** 10.1093/nargab/lqag128

**Published:** 2026-10-21

**Authors:** Yiyi Cheng, Toby Kettlewell, Ross F Laidlaw, Olympia M Hardy, Andrew McCluskey, Thomas D Otto, Domenico Somma

**Affiliations:** School of Infection & Immunity, University of Glasgow, G12 8TA,Glasgow, United Kingdom; Bernhard Nocht Institute of Tropical Medicine, 20359,Hamburg, Germany; School of Mathematics & Statistics, University of Glasgow, G12 8QQ,Glasgow, United Kingdom; School of Infection & Immunity, University of Glasgow, G12 8TA,Glasgow, United Kingdom; Center for Infectious Diseases, Leiden University, 2300 RC,LUMC, Leiden, Netherlands; School of Infection & Immunity, University of Glasgow, G12 8TA,Glasgow, United Kingdom; School of Infection & Immunity, University of Glasgow, G12 8TA,Glasgow, United Kingdom; Bernhard Nocht Institute of Tropical Medicine, 20359,Hamburg, Germany; School of Infection & Immunity, University of Glasgow, G12 8TA,Glasgow, United Kingdom; Bernhard Nocht Institute of Tropical Medicine, 20359,Hamburg, Germany; University of Hamburg, 20148,Hamburg, Germany; LPHI, CNRS, INSERM, Université de Montpellier, 34095,Montpellier, Occitanie, France; School of Infection & Immunity, University of Glasgow, G12 8TA,Glasgow, United Kingdom; Bernhard Nocht Institute of Tropical Medicine, 20359,Hamburg, Germany; Division of Rheumatology and Clinical Immunology, Università Cattolica del Sacro Cuore, 00168,Rome, Italy

## Abstract

Accurate identification of differentially expressed genes (DEGs) in single-cell RNA sequencing data remains challenging. Single-cell-specific statistical models often report large numbers of candidate genes but can exhibit inflated false positive rates, whereas pseudobulk approaches improve false discovery control at the cost of reduced sensitivity. To overcome the noise and bias that other tools have, and allow the user to have more control of the DEG process, we present CellDEEP, which uses a cell aggregation (metacell) approach. This tool provides a framework for flexible selection of pooling strategies and parameterization for differential expression analysis. Benchmarking on simulated and real datasets, including COVID-19 and rheumatoid arthritis, shows that CellDEEP often outperforms other methods, consistently reduces false positives compared to single-cell methods and recovers more true positives than pseudobulk methods. Our work shifts the focus from selecting a single ‘best’ method to an approach that reduces cell-level noise while preserving biological signal, together with transparent validation framework, advancing more reliable differential-expression analysis in single-cell transcriptomics.

## Graphical Abstract

**Graphical Abstract:**
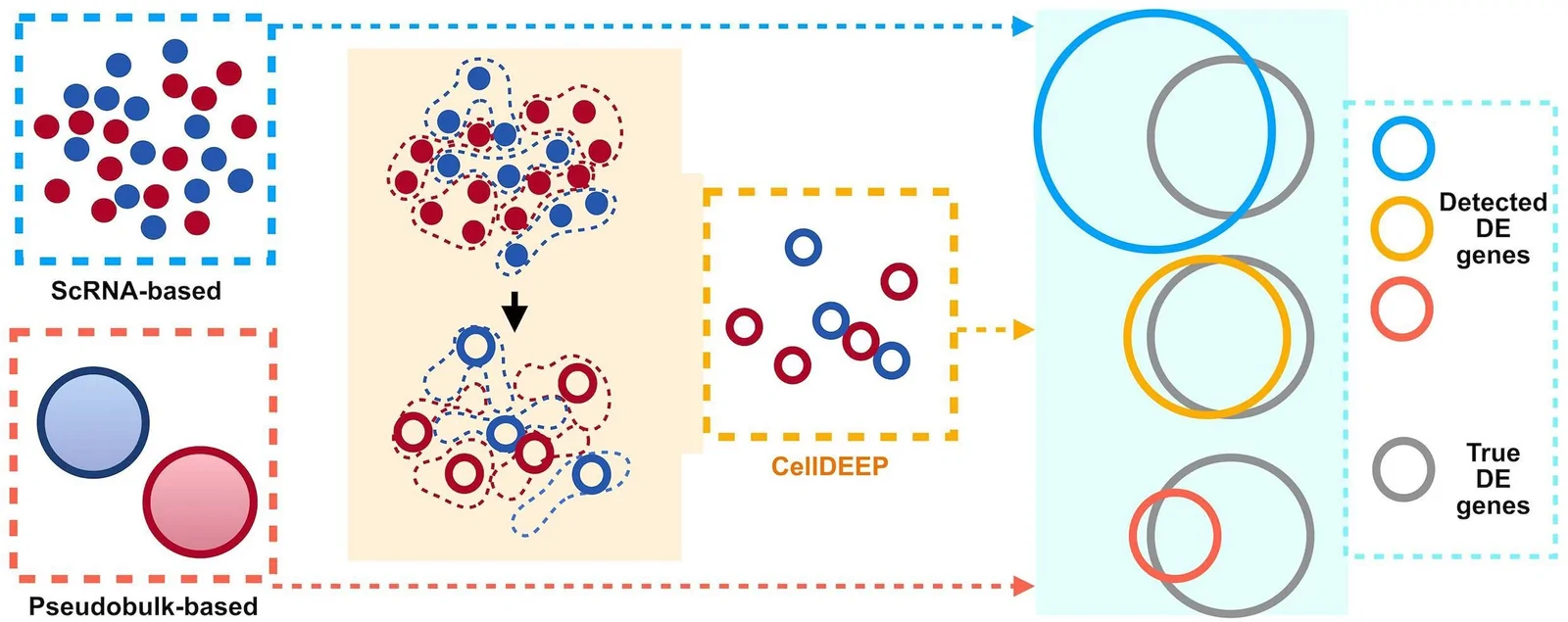

## Introduction

RNA sequencing (RNA-seq) has transformed transcriptomic studies, enabling the exploration of gene expression across tissues and conditions. Bulk RNA-seq, which measures average expression across pooled cells, has been instrumental in disease classification and response prediction, but it obscures cell-specific dynamics by averaging expression across diverse cell types. In 2009, single-cell RNA sequencing (scRNA-seq) emerged [1] and gained traction as the cost of high-throughput platforms declined. It enables the detection of rare cell types, lineage tracing, and gene regulation [2]. However, this power comes at a cost: scRNA-seq data are inherently sparse, with high dropout rates and zero-inflation, in which genes may be undetected despite being expressed, posing significant challenges for differential expression (DE) analysis [3, 4].

Established differential-expression (DE) methods for bulk RNA-seq, such as DESeq2 [5], edgeR [6], and limma [7], model expression using statistical distributions (e.g. negative binomial) and can account for complex experimental designs through generalized linear models. For scRNA-seq, these methods are often applied using a pseudobulk approach, where expression counts are aggregated across cells from the same condition and sample [8]. While this approach improves statistical power and controls false positives, it sacrifices cell-level resolution.

Alternative scRNA-seq-specific methods such as MAST [9] model dropout explicitly and retain single-cell resolution, offering improved sensitivity in detecting subtle gene-expression changes. Yet these methods can inflate false positive rates (FPR), particularly when the assumptions about data distribution do not hold [10].

The performance of DE methods varies widely across benchmarking studies. Pseudobulk methods are generally preferred for their robustness [4, 11], while scRNA-based methods offer higher sensitivity in contexts where capturing subtle differences is crucial [12]. It has also been proposed that the DE strategy should be tailored to the study design: for case-control studies, pseudobulk methods are preferable, whereas for intra-condition comparisons (e.g. developmental trajectories), scRNA-based methods may be more suitable [13].

Recently, linear mixed models for scRNA-seq data have been developed. While promising, it is unclear how widely they are adopted, and they face practical challenges: benchmarks indicate they do not consistently outperform the simpler, well-established pseudobulk approaches, and they can be computationally intensive [11, 14].

Motivated by these limitations, we developed CellDEEP (Cell DiffErential Expression by Pooling), a hybrid DE analysis framework that aims to combine the strengths of both approaches. By selectively pooling cells prior to DE testing, CellDEEP reduces noise and zero inflation while retaining resolution and biological signal. In this study, we benchmark CellDEEP against scRNA-based and pseudobulk-based DE methods, using both simulated and real-world datasets, evaluate the effects of pooling strategies and data quality, and provide practical guidance for DE analysis in scRNA-seq studies.

## Methods

### CellDEEP architecture

CellDEEP is designed to merge single cells into metacells before DE analysis to reduce the likelihood of false positives in the standard scRNA-seq DE analysis pipeline. The complete pipeline consists of three main steps: dataset preprocessing, metacell creation, and DE analysis.

Dataset preprocessing extracts three key identifiers required for downstream analysis: group ID, sample ID, and cluster ID.

The metacell creation workflow consists of three main steps: (i) defining subset datasets, (ii) selecting cells for pooling, and (iii) aggregating gene read counts. Cells are first separated into multiple subset datasets based on shared cluster, group, and replicate labels. Each cluster *k*, group *g*, and replicate *r*, we define


\begin{eqnarray*}
{{X}_{k,g,r}} = \left\{ {c_i^{\left( {k{\mathrm{,}}g{\mathrm{,}}r} \right)}\mid i = 1,\ldots ,{{N}_{k,g,r}}} \right\},
\end{eqnarray*}

where $c_i^{( {k{\mathrm{,}}g{\mathrm{,}}r} )}$ denotes the barcode of the *i*-th cell in that subset and ${{N}_{k,g,r}}$ is the number of cells in the subset.

From each subset ${{X}_{k,g,r}}$, CellDEEP selects *n* cells to pool and aggregates their gene read counts to construct metacells. The pooling size *n\ * is user-defined, with a minimum of 1. If a subset contains fewer than *n* cells, it is discarded.

### Selecting cells to pool from subset

For cell selection, we implemented two strategies: random selection and k-means clustering selection.

Assuming $n\, < \, {{N}_{k,g,r}}$, for random selection we randomly select *n* cells from each ${{X}_{k,g,r}}\ $ can pool the cells. The *n* cells will form one metacell in further aggregation and are then removed from ${{X}_{k,g,r}}$. Sampling is then repeated for the next *n* remaining cells until all cells in ${{X}_{k,g,r}}\ $ have been processed. The final metacell will contain the remaining cells.

For k-means clustering selection, the number of clusters, *K*, is set according to the intended pooling size *n* and ${{N}_{k,g,r}}$:


\begin{eqnarray*}
K = \left\lfloor \frac{{{{N}_{k,g,r}}}}{n}\right\rfloor + 1.
\end{eqnarray*}

If $K\ \le \ 2$, the procedure is not applied because the subset contains too few cells for meaningful clustering (in which case a smaller *n* is required). Otherwise, *k*-means clustering is performed on the variance-standardized PCA embedding of ${{X}_{k,g,r}}$ to obtain *K* clusters. Cells within each cluster are then merged to form one metacell per cluster.

### Aggregating gene read counts

For each pool of *n\ * selected cells from subset ${{X}_{k,g,r}}$, CellDEEP aggregates gene read counts to construct a metacell.

Let ${{y}_{ij}}$ denote the read count of gene *j* in cell ${{c}_i}$.

For a pool $P \subset {{X}_{k,g,r}}$ with $\mid P\mid = n$, aggregated counts for gene *j* are computed as


\begin{eqnarray*}
{\mathrm{sum:}}{{Y}_j} = \sum \limits_{{{c}_i}\in P} {{y}_{ij}},{\mathrm{\ mean:}}{{Y}_j} = \frac{1}{n}\sum \limits_{{{c}_i}\in P} {{y}_{ij}}.
\end{eqnarray*}

The resulting vector, $Y = ( {{{Y}_1},\ldots,{{Y}_G}} )$, defines the read count profile of one metacell, *G* is the number of genes. ${{X}_{k,g,r}}$ Pooling and aggregation are repeated until all cells in subset are processed.

This process is applied to all subset datasets, the resulting metacell vectors are then combined to construct a complete metacell expression matrix. Finally, a pooled Seurat object is constructed from the aggregated metacell matrix.

### Application of DE methods

DE analysis in CellDEEP follows the standard Seurat [15] workflow and is performed using the *FindMarkers* function. All parameters are configurable as in the standard Seurat pipeline, with a default *p.adj* (adjusted *P*-value) threshold of 0.05 and *logfc.threshold* of 0.25. To ensure comparability between MAST and DESeq2, for the receiver operating characteristic (ROC) curve, precision-recall curve (PRC), and null hypothesis *P*-value test we removed all filters, hence using the full gene list. For pseudobulk-based methods we used DESeq2 and limma-voom, through *pbDS()* function from Muscat [4] Version 1.26.0 package (with default parameters *p_adj.loc* < 0.05; *min_cells*=1). Before DE analysis, the Seurat object is converted to the *SingleCellExperiment* object, and then the pseudobulk data matrix is generated by summing gene read counts across cells from the same group, cluster, and biological replicates. The design matrix, which is used in DE comparison, is calculated by the combination of ‘*cluster_id*’ and ‘*sample_id*’. All processing steps were carried out using the standard Muscat pipeline.

For CellDEEP, we also used MAST and DESeq2 from Seurat package v. 4.3.0 as analysis methods after pooling.

### Simulation

To evaluate the performance and utility of CellDEEP, we first tested it on simulated datasets. We used the simulation framework from two published simulation pipelines: Crowell *et al*. [4] and Zimmerman *et al*. [3]. To maximize the similarity of the simulated dataset, we used the same input settings for both frameworks (indicated respectively as Muscat and Zimmerman): we generated datasets with 4000 cells, 2000 genes, 2 groups, and each group with 10 replicates, and 20% DE genes. For Muscat, five reference datasets were used: the original reference dataset from Zimmerman (Alpha cells from pancreas) [3] and Muscat [4] (PBMC from Lupus patients obtained before and after 6 h-treatment with IFN-β), CD14^+^ monocytes and CD8^+^ T cell (in healthy and severe COVID-19 patients) [16], and TREM2^high^ macrophage cluster (from rheumatoid arthritis active patients and healthy donors) [17]. The above datasets were also used for the Zimmerman pipeline with the exclusion of the PBMC dataset from Lupus patients as it failed to meet the framework requirements.

In particular, for Muscat framework (Muscat v. 1.26.0) we simulated a total of 10 datasets. For each reference dataset, we generated two simulation settings with average log_2_ fold changes (log_2_FC) of 2 and 3, respectively.

Muscat implements multiple DE patterns, including changes in the mean expression (DE), changes in the proportions of cells expressing a gene (DP), changes in differential modality (DM), and changes in both proportions and modality (DB). Genes that are not subject to changes are either equivalently expressed (EE) or expressed at low and high levels in equal proportions (EP) across conditions. Full details of these simulation parameters are provided in the original Muscat publication [4]. To maintain comparability between the two simulation frameworks, the main Muscat simulations used 20% DE, 0% DP, 0% DM, 0% DB, 40% EE, and 40% EP genes across all 10 datasets.

In addition to the main Muscat simulation described above, we performed three complementary Muscat-only simulation analyses to further evaluate CellDEEP. We first defined a weighted-mixed setting consisting of 15% DE, 2% DP, 2% DM, 1% DB [4, 18], 40% EE, and 40% EP genes. Using this setting, we generated datasets from the same five reference datasets at log_2_FC values of 1, 2, and 3, resulting in 15 additional datasets.

For the Zimmerman simulation instead, we used the Murphy & Skene adaptation of the original Zimmerman framework to jointly evaluate type I and type II error [19]. Simulations were performed using the *simulate_hierarchicell* function. For each reference dataset, a non-DE matrix and a DE matrix were generated separately using identical simulation settings and then were combined into a single expression matrix. Genes from the DE matrix were treated as ground-truth DE genes, and genes from the non-DE matrix were treated as non-DE genes. The combined matrix was then converted into a Seurat object. For each reference, we simulated two datasets with FC of 4 and 8 separately, to match log_2_FC used in Muscat, for a total of eight datasets. In total, we generated 33 simulated datasets: 10 from Muscat (DE-only), 15 from Muscat (mixed-weighted), and 8 from Zimmerman. These datasets were derived from five distinct real reference datasets. A detailed summary of all simulation parameters for each framework and scenario is provided in Supplementary Tables S6 and S7.

### Simulation analysis and result evaluation

We evaluated DE methods on all simulated datasets using three categories of approaches: (i) single-cell methods (DESeq2, MAST) applied directly to the original data; (ii) CellDEEP with all parameter combinations (pool size, cell selection: random or k-means, aggregation: mean or sum) followed by DESeq2 or MAST; and (iii) pseudobulk methods (DESeq2, limma).

Method performance was assessed using counts of true positives (TP), false positives (FP), true negatives (TN), and false negatives (FN), from which we computed precision, sensitivity, accuracy, and F1 score.

To test whether performance differences between methods were statistically significant, we used a paired Wilcoxon signed-rank test (*wilcox.test* function from the R stats package, with paired = TRUE and default parameters).

### ROC and PRC analysis

ROC and PRC analysis requires a ranked full gene list as input.

For scRNA and CellDEEP, full gene lists were generated using the same DE analysis process with *logfc.threshold = 0 and min.pct = 0*, and no additional *P*-value or logFC filtering. For pseudobulk analyses, full lists were generated by same DE process without additional gene filtering.

To rank the gene list, a ranking score *π * [20] was calculated for each gene *j*:


\begin{eqnarray*}
\pi = \ - \mathrm{ lo}{{\mathrm{ g}}_{10}}\left( {{{p}_j}} \right) \times \ \left| {\mathrm{ lo}{{\mathrm{ g}}_2}\mathrm{ F}{{\mathrm{ C}}_j}} \right|,
\end{eqnarray*}

where *p* is the raw *P*-value. Dataset-specific ROC curves were generated using the roc function from the R package *pROC*, and dataset-specific PRC were generated using the *pr.curve* function from the R package *PRROC*. AUROC and AUPRC were calculated from the same functions, respectively.

To generate mean curves, dataset-specific ROC and PRC were interpolated onto common grids from 0 to 1 at 0.01 intervals. Mean ROC curves were obtained by averaging true-positive rates at each false-positive-rate point, and mean PRC were obtained by averaging precision values at each recall point. AUROC and AUPRC were calculated separately for each dataset and summarized across simulations.

### Dropout rate

Dropout rate was defined following design of Zimmerman [3]. For each gene *g*, the dropout rate ${{D}_g}$ was calculated as the proportion of cells with zero read counts among all cells.


\begin{eqnarray*}
{{D}_g} = \ \frac{1}{N}\mathop \sum \limits_{i = 1}^N 1\left( {{{x}_{gi}} = 0} \right),
\end{eqnarray*}

where *N* represents the total number of cells and ${{x}_{gi}}$ represents the read count of gene *g* in cell *i*. $1( {{{x}_{gi}} = 0} )$ is an indicator function that equals to 1 if ${{x}_{gi}}$ = 0 and 0 otherwise.

### Published dataset test

Two published datasets are used in both simulation (as reference) and real dataset tests. To maximize similarity, we preprocess them in the way described below.

### COVID-19 PBMC

The h5ad file was downloaded from https://covid19cellatlas.org/ ‘COVID-19 PBMC Ncl-Cambridge-UCL’—Haniffa Lab, and transformed into a Seurat object with the *Convert* function. For the COVID-19 dataset: CD8^+^ T cells, B cell, CD14^+^ monocytes, and NK cells CD56^high^ cluster were selected for testing. Further details of datasets can be found in Supplementary Table S1. For each selected cluster, we subset them from the original COVID-19 data, DE analysis between healthy and severe COVID-19 patients was then performed. Preprocessing consists of *NormalizeData, ScaleData, RunPCA* with default parameters and 30 dimensions from Seurat package for k-means cell selection. DE analysis between healthy and severe COVID-19 patients was then performed.

### Rheumatoid arthritis

The RDS object was provided upon request from the corresponding research group [17], TREM2^high^ and HLA^high^CLEC10A^high^ clusters were selected for downstream analysis. Data details are provided in Supplementary Table S2. Preprocessing includes *NormalizeData, ScaleData, RunPCA* with 30 dimensions from the Seurat package for k-means cell selection. DE analysis was then performed, with comparisons between ‘Healthy’ and ‘Active RA’.

### Real datasets evaluation

Two downstream analyses were performed to evaluate method performance: Null hypothesis *P*-value test for FP rate (FPR) and gene-ontology (GO) enrichment analysis for pathway recovery rate (PRR) and signal density. We used the R package clusterProfiler v. 4.6.2 to perform GO terms enrichment analysis on identified DE genes (GO terms set as ‘ALL’).

### Null hypothesis *P*-value histogram

Null hypothesis data were generated by aggregating biological replicates from the same biological condition (e.g. healthy) into two separate virtual groups. For each cluster, the condition with most replicates was split in two groups, applying DE analysis between these two groups (Supplementary Table S3). A gene list with *P*-values were generated after DE analysis. Under the null hypothesis, the *P*-value of all genes should be uniformly distributed over [0,1], therefore a 5% FPR is expected. Genes with *P*-value <.05 are considered as FP results.

To evaluate method performance, the FPR was calculated by dividing the number of false positive genes by the total number of genes in the corresponding null hypothesis dataset.

### Gene Ontology analysis

To assess TP detection on real data, we predefined, for each disease context, a set of GO terms expected to be enriched based on established biological knowledge. All selected GO term sets are provided in Supplementary Table S4.

For each method, we quantified TP performance using two metrics: PRR and signal density. Let *G* denote the selected GO term list, *D\ * represents the set of GO terms detected by a method, and ${{N}_{\mathrm{ DE}}}$ the number of DE genes detected by that method.

PRR measured the proportion of expected GO terms recovered, defined as


\begin{eqnarray*}
\mathrm{Pathway}\ \mathrm{Recovery}\ \mathrm{Rate} = \ \frac{{\left| {D\ \cap G} \right|}}{{\left| G \right|}}.
\end{eqnarray*}

Signal density measured the number of recovered expected GO terms per detected DE gene, and thus reflecting precision in capturing biologically relevant signal rather than increased DE gene calls, defined as


\begin{eqnarray*}
\mathrm{Signal}\ \mathrm{Density} = \ \frac{{\left| {D\ \cap G} \right|}}{{{{N}_{\mathrm{ DE}}}}}.
\end{eqnarray*}

For the COVID-19 PBMC data, we focused on cell types involved in antiviral defence: antigen-presenting cells (monocytes CD14^+^, B cells) and effector cells (CD8^+^ T cells, NK cells CD56^high^) that respond to virus by activating innate immunity pathways and mechanisms. For these, we selected 91 related GO terms capturing key innate immune processes, including antiviral responses, interferon signalling, antigen processing and presentation, and inflammatory pathways.

For RA datasets, we defined two separate true positive sets: (i) for genes upregulated in healthy synovial tissue, we selected 23 GO terms related to normal tissue homeostasis; and (ii) for genes upregulated in active RA, we selected 46 GO terms associated with chronic inflammation, including antiviral and interferon responses, antigen presentation, and chemokine activity, consistent with established literature on RA pathogenesis [21]. For the HLA^high^ cluster, where healthy baseline biology is less clearly defined, we focused exclusively on genes upregulated in active RA, selecting 105 GO terms related to viral response, antigen presentation, and MHC-related processes.

### Statistical reporting

For real data analyses, exact sample sizes are detailed in Supplementary Tables S1–S3. For simulation studies, exact sample sizes and parameters are detailed in Supplementary Tables S6 and S7. Performance metrics (accuracy, precision, sensitivity, F1-score, AUROC, and AUPRC) were summarized as medians with interquartile ranges.

## Results

We developed CellDEEP, an R package to improve DE process by pooling single cells into metacells. CellDEEP aggregates cells within user-defined clusters into metacells. The method offers several user-configurable parameters: (i) cells can be selected for pooling either randomly within a cluster or based on k-means clustering in the embedding space (Supplementary Fig. S1); (ii) UMI counts for the metacell can be generated by summation (‘sum’) or averaging (‘mean’); and (iii) the number of cells per metacell is adjustable. The resulting metacell count matrices can then be analysed using established DE tools such as DESeq2 or MAST.

### Exploring the best parameters set for CellDEEP

To identify optimal parameter settings, we first evaluated how count matrix generation methods (sum versus mean) and cell selection strategies (k-means versus random) affect performance using two established simulation frameworks: Muscat and Zimmerman (see the ‘Methods’ section), comprising 18 simulated datasets with diverse cell types and experimental conditions.

The most substantial performance difference was observed between count aggregation methods, where, across both simulation frameworks, sum consistently outperformed mean in accuracy and sensitivity (Fig. 1A and B, and Supplementary Fig. S2A). This effect was most pronounced in the Zimmerman simulation with MAST, where sum outperformed mean by 7 percentage points in accuracy (*P* ≤ .08).

**Figure 1.**
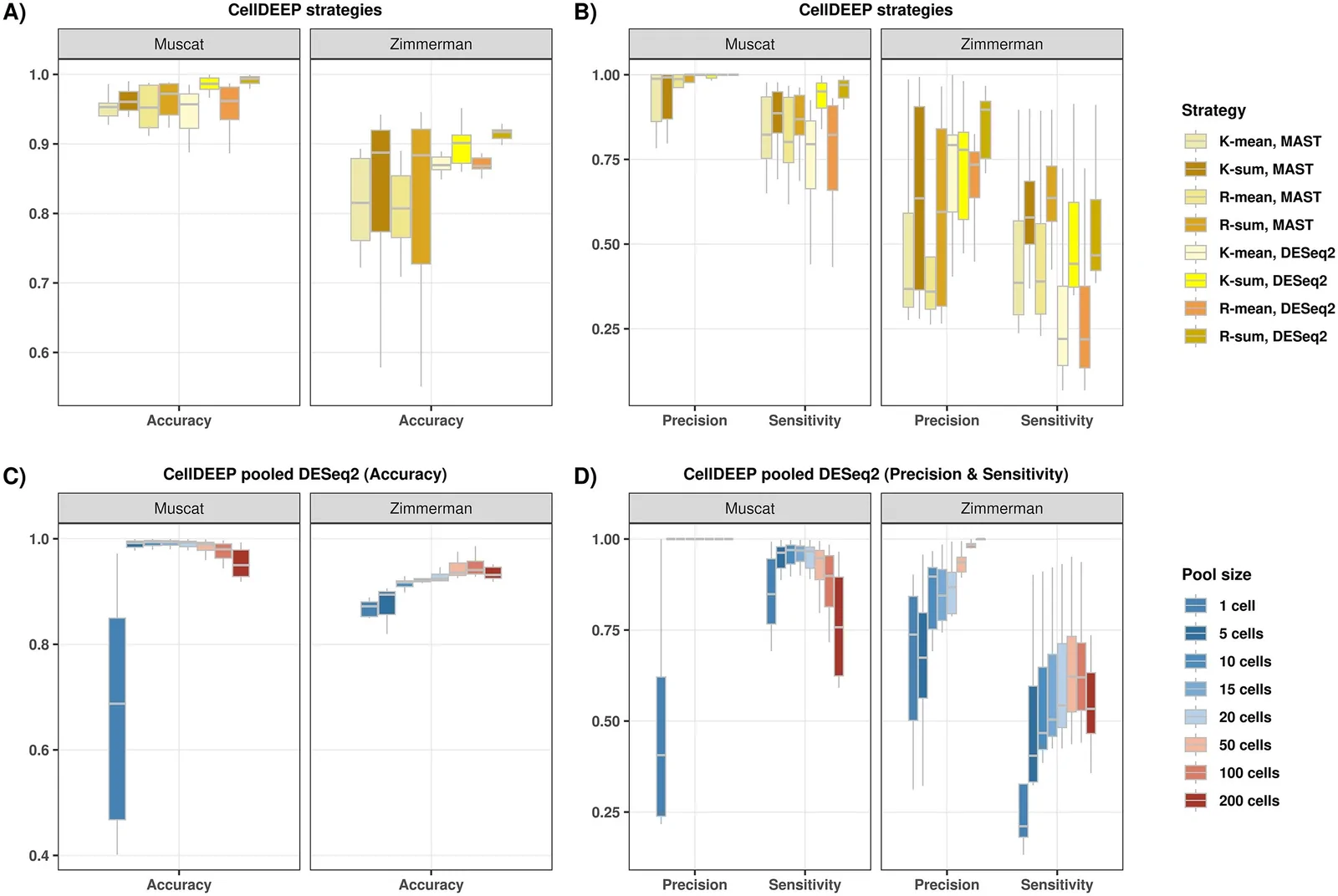
CellDEEP strategy performance comparison in simulated datasets. (**A, B**) Comparison of accuracy, precision, and sensitivity across both Muscat (only DE genes) and Zimmerman simulation frameworks. K: cell selection by k-mean clustering; R: random cell selection; sum: aggregating read counts by summing; mean: aggregating read counts by averaging. (**C, D**) Effect of metacell size on accuracy, precision, and sensitivity, using optimal parameters (random selection, sum aggregation, DESeq2), in both Muscat and Zimmerman simulation framework. Metacell sizes tested: 1 (single-cell), 5, 10, 15, 20, 50, 100, and 200 cells.

In contrast, our analysis revealed that the choice of cell selection strategy (k-means versus random) had a minimal impact on performance, with a non-significant difference in accuracy of only 1–2 percentage points (Table 1).

**Table 1.** Comparison of CellDEEP strategies for accuracy in the Zimmerman simulation

		Accuracy	
Aggregation parameter	Cell selection parameter	MAST	DESeq2
Mean	Kmean	0.82	0.87
	Random	0.81	0.87
Sum	Kmean	0.89	0.90
	Random	0.88	**0.92**

Read-count aggregation methods (Mean versus Sum) and cell-selection approaches (K-means versus Random) are evaluated over two DE methods. Values represent the median accuracy across 10 simulated Zimmerman datasets.

The best results in terms of accuracy (0.92) were obtained by using a random selection strategy, summing the read counts and using DESeq2 (R-sum, DESeq2). These trends were consistently observed in the Muscat simulation framework as well (Supplementary Table S5).

To better understand the drivers of accuracy, we also assessed precision and sensitivity separately (Fig. 1B and Supplementary Fig. S2A). This revealed that sensitivity was strongly dependent on aggregation method, with sum yielding significantly higher values than mean (*P* < .03). For instance, in the Zimmerman simulation, using random selection with DESeq2, the sum aggregation (R-sum, DESeq2) achieved median precision and sensitivity of 0.90 and 0.47, respectively, whereas the mean aggregation under the same conditions (R-mean, DESeq2) yielded lower values of 0.73 and 0.22.

Based on these results, we conclude that sum aggregation consistently outperforms mean in both accuracy and sensitivity. The homogeneity of cell clusters within these simulated datasets appears to limit the potential for selection strategies to yield significant performance gains. Our analysis also highlighted a clear influence of the simulation framework itself, with Muscat yielding higher overall precision and sensitivity than Zimmerman across all parameter combinations.

### The effect of pooling cells to metacells

After establishing optimal parameters, we evaluated the effect of metacell size on detection accuracy. For each simulation we generated 4000 cells, and we varied the number of cells pooled per metacell while maintaining optimal parameters (R-sum, DESeq2). We tested extreme cases, ranging from single-cell (standard scRNA-seq) to large pools of 200 cells. While results differed slightly between simulation frameworks, they revealed consistent pattern: accuracy improved as more cells were pooled, reached an optimum, and then declined with the largest pool sizes (Fig. 1C and Supplementary Fig. S2B). In the Zimmerman simulations, accuracy increased incrementally from 0.87 (single-cell) to a peak of 0.94 with 100 cells per metacell, before decreasing slightly to 0.93 with 200 cells. The Muscat simulations showed a different trajectory: accuracy rose sharply from 0.75 (single-cell) to ~0.99 with moderate pool sizes (5–20 cells), where it remained stable, before to falling to 0.94 at 200 cells.

In both simulations, the lowest accuracy was observed with single-cell analysis (no pooling), corresponding to conventional scRNA-seq methods (Fig. 1C and Supplementary Fig. S2B). Accuracy improved with pooling, reaching an optimum at 20 cells for Muscat and 100 cells for Zimmerman, before declining with the largest pool sizes.

To investigate this further, we complemented our accuracy analysis with precision and sensitivity, revealing the drivers of this pattern (Fig. 1D and Supplementary Fig. S2B), as both were strongly affected by pool size. In Zimmerman simulations, precision increased progressively, gaining ~20 percentage points from pools of 5–200 cells. In Muscat simulations, precision rose sharply from 0.75 (single-cell) to a near-perfect 1.0 with any level of pooling. In general, by pooling sufficient cells, we can achieve a precision of 1 or close to it.

Sensitivity instead followed a different trajectory. In Zimmerman simulations, sensitivity peaked at 0.62 with 50 cells, followed by a decline to 0.53 at 200 cells. Muscat simulations followed a similar pattern of increase and then decrease, though the specific values and peak location differed. This decline in sensitivity with the largest pools explains the corresponding drop in overall accuracy.

In summary, both frameworks exhibited dramatic improvements when metacells were used compared to single-cell analysis. These results demonstrate that metacell pooling substantially enhances scRNA-seq analysis for both simulation frameworks. The gains in precision and sensitivity, at least at intermediate pool sizes for Muscat, highlight the utility of metacells in balancing statistical power and resolution. This approach mitigates noise inherent in single-cell data while avoiding the oversimplification of full-cluster aggregation, where sensitivity is lost. On the other hand, in practice, the maximum feasible pool size is constrained by the minimum number of cells per cluster available across samples to be pooled. Setting the pool size too high risks excluding samples with lower cell counts, which could reduce statistical power. Therefore, while our simulations show that pooling improves accuracy, the choice of pool size for a given study should balance these accuracy gains against the risk of sample loss.

### Comparative performance of CellDEEP against scRNA-seq and pseudobulk methods

After exploring how to parameterize CellDEEP, we next evaluated the performance of CellDEEP against conventional single-cell (DESeq2, MAST) and pseudobulk approaches (DESeq2, limma). While performance trends were consistent across simulation frameworks, specific outcomes exhibited framework-dependent variations. Based on the previous section, we decided to use 20 as the number of cells to pool.

As shown previously in the simulations generated by the Zimmerman framework, CellDEEP (R-sum, DESeq2) demonstrated a 5-percentage-point increase in accuracy over standard scRNA-seq DESeq2. Furthermore, CellDEEP outperformed MAST by 11 percentage points. A similar trend was observed in the simulations generated by Muscat (Table 2).

**Table 2.** Accuracy (median) of DE approaches across two simulation frameworks

		Sc		Pseudobulk	
Simulation framework	CellDEEP DESeq2(R-sum)	DESeq2	MAST	DESeq2	limma
Zimmerman	**0.92**	0.87	0.81	0.91	**0.92**
Muscat	**0.99**	0.69	0.96	**0.99**	0.98

Comparing CellDEEP (Random–Sum–DESeq2), scRNA-seq (DESeq2, MAST), and Pseudobulk (DESeq2, limma).

In comparison with pseudobulk methods, CellDEEP achieved very similar results in terms of overall accuracy (Table 2, Fig. 2A, and Supplementary Fig. S2C). However, its performance in precision and sensitivity was simulation framework dependent. While CellDEEP matched pseudobulk accuracy in Muscat simulations, results from the Zimmerman framework revealed distinct trade-offs (Supplementary Fig. S3): CellDEEP maintained higher sensitivity (0.61) compared to both pseudobulk DESeq2 and limma (0.54). Conversely, CellDEEP showed moderately lower precision (0.83) than limma (0.91) and pseudobulk DESeq2 (0.88).

**Figure 2.**
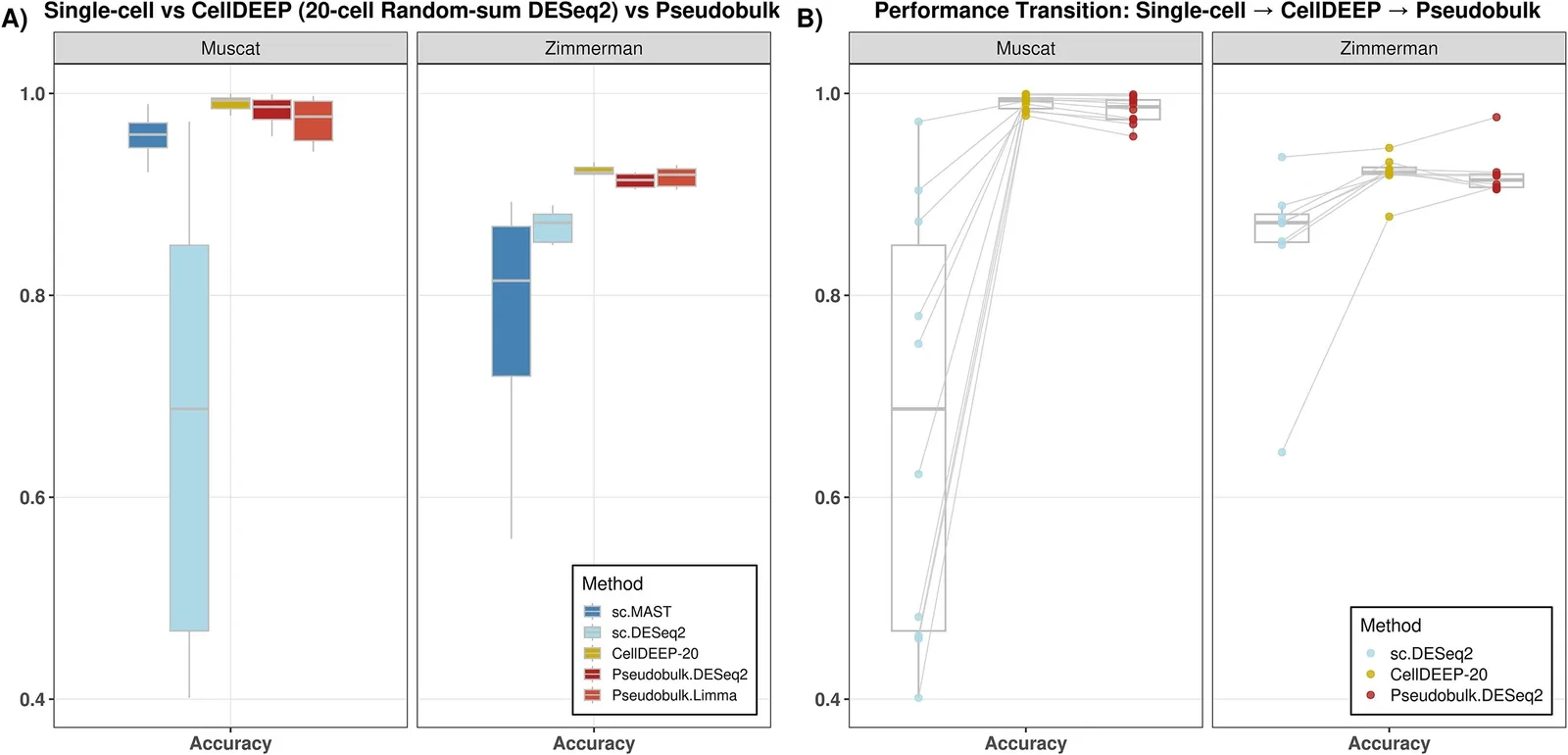
CellDEEP performance evaluation with scRNA-based and pseudobulk-based methods in simulated datasets. (**A**) Accuracy of DE approaches across two simulation frameworks, comparing CellDEEP (Random–Sum–DESeq2), scRNA-seq (DESeq2, MAST), and Pseudobulk (DESeq2, limma). (**B**) Accuracy changes across same dataset (connected by lines) with different DE analysis methods, in two simulation frameworks comparing CellDEEP (Random–Sum–DESeq2), scRNA-seq (DESeq2), and Pseudobulk (DESeq2). Each line represents a dataset.

This trade-off is further reflected in the mean ROC and PRC (Supplementary Fig. S4). CellDEEP achieved a median AUROC of 0.990 and a median AUPRC of 0.984, comparable to pseudobulk DESeq2 and limma (median AUROC: 0.993 and 0.991; median AUPRC: 0.986 and 0.983, respectively), and substantially higher than scRNA-seq methods (median AUROC: 0.965 for sc.MAST and 0.930 for sc.DESeq2; median AUPRC: 0.949 and 0.855, respectively). These results confirm that CellDEEP achieves near-pseudobulk performance in ranking and classification ability, while offering improved sensitivity.

Our simulation results demonstrate that CellDEEP reliably and substantially outperforms standard single-cell analysis methods, likely due to a reduction of technical noise and dropout effects (Supplementary Fig. S5). In comparisons with pseudobulk methods, however, the performance difference was smaller and more variable. CellDEEP achieved higher accuracy than pseudobulk in 14 out of 18 simulated datasets (Fig. 2B). This variability, even within controlled simulations, highlights that DE analysis is inherently dataset dependent.

These findings show that CellDEEP outperforms scRNA-seq and is as good as pseudobulk on simulated data. However, simulation may favour pseudobulk methods, as it cannot capture real noise. Therefore, evaluation using real-world data is necessary to provide a more realistic assessment.

### CellDEEP outperforms both pseudobulk- and scRNAseq-based methods on real datasets

To evaluate CellDEEP’s performance on real biological data, we analysed multiple immune cell clusters from two public datasets: a COVID-19 PBMC dataset and a RA synovial tissue dataset.

Our evaluation strategy was based on two principles: (i) assessing false positives by testing under the null hypothesis, and (ii) evaluating true positive detection by examining expected GO terms and their associated genes from well-established biological processes. We used two papers well-established in the field: for COVID-19, we analysed antigen-presenting cells (CD14^+^ monocytes, B cells) and antiviral effector cells (plasmacytoid DCs, CD8^+^ T cells, NK cells CD56^high^) from healthy versus severe patient samples; for rheumatoid arthritis (RA), we analysed synovial macrophages, which play a key role in immune response and tissue homeostasis in the RA synovial membrane, and compared them with healthy controls [17, 22, 23]. For these analyses, we used a metacell pool size of 10 cells to ensure that sufficient metacell replicates could be generated from all clusters, including those with limited per-sample cell counts (e.g. NK cells CD56^high^).

### False positive rate

To estimate the FPR, we performed negative control comparisons where no DE should exist [24]. This was achieved by splitting the replicate samples from a single biological condition (e.g. healthy donors) into two artificial groups. Under these conditions, genes with a *p.adj* <.05 after DE were classified as false positives.

Overall, MAST exhibited the poorest performance across single-cell (FPR 0.3–0.6), compared to MAST with CellDEEP (R-sum) (0.12–0.55), and CellDEEP (R-mean) (0.08–0.16) configurations (Fig. 3A). In our experiment and tested dataset, MAST failed to control false positives well compared to other scRNA-seq methods. Both scRNA-seq DESeq2 and pseudobulk limma also exceeded the 0.05 false positive threshold. The two best-performing tools were pseudobulk DESeq2 (FPR ≤ 0.02) and CellDEEP (R-mean, DESeq2) (FPR ≤ 0.03), both of which maintained false discovery rates below the 0.05 cutoff. Results were consistent across individual datasets, with CD8^+^ T cells presenting the greatest challenge (FPR range 0.01–0.6) and B cells the least (FPR range 0.01–0.3).

**Figure 3.**
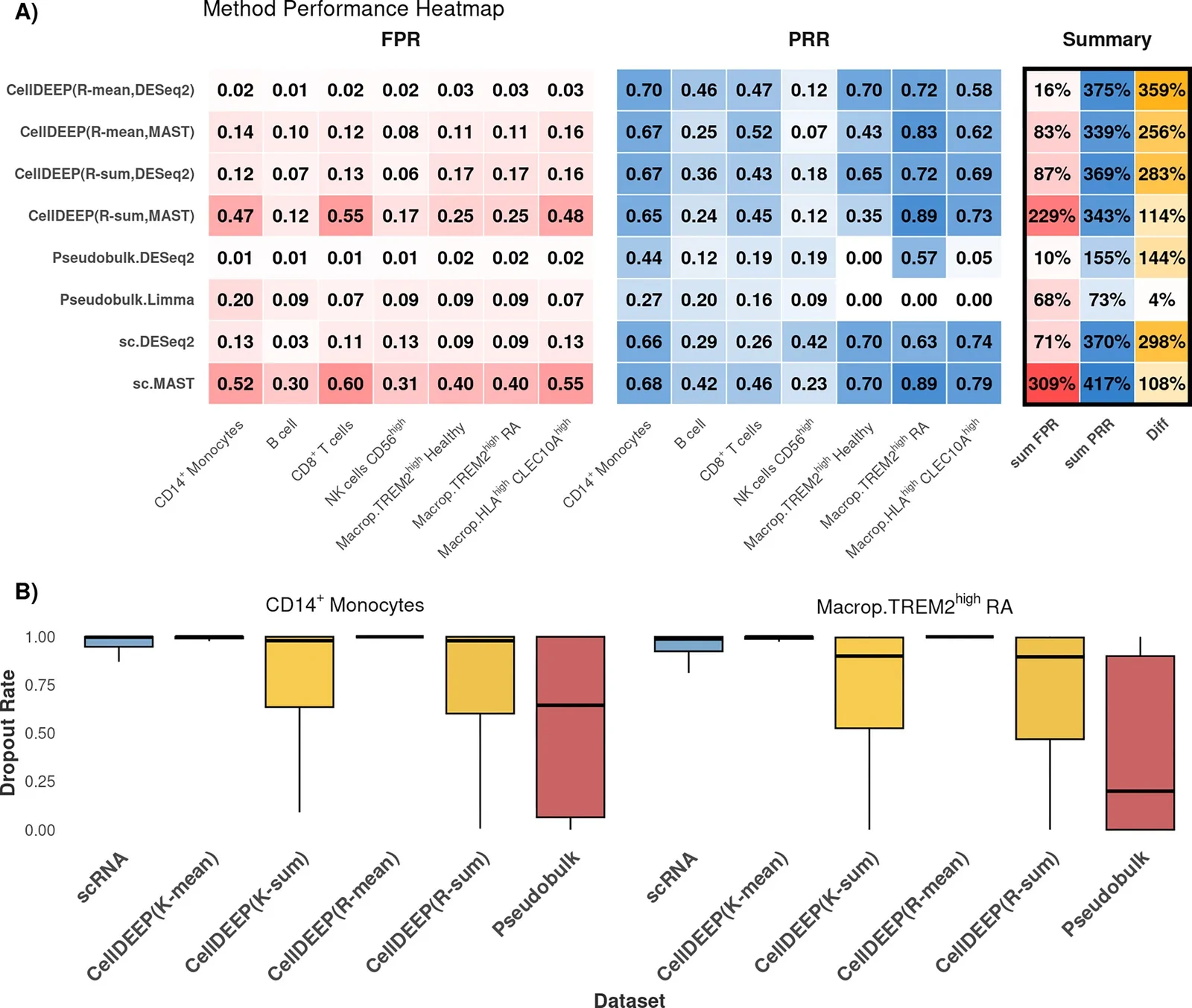
Method evaluation on published datasets: FPR and PRR. (**A**) Performance summary across all datasets. For each method, we calculated FPR from negative control comparisons (same-condition replicate splits); and PRR as the proportion of curated true positive GO terms detected. Sum FPR and Sum PRR represent totals across all datasets (percentages). Diff (Sum PRR − Sum FPR) indicates the net balance between true positive detection and false positive control. Methods evaluated: scRNA-seq (MAST, DESeq2); pseudobulk (DESeq2, limma); CellDEEP (random selection, 10 cells per metacell) with sum/mean aggregation and DESeq2/limma. (**B**) Gene dropout comparison for CD14^+^ monocytes (COVID-19) and TREM2high macrophages (RA). For each gene, dropout rate was calculated as the proportion of cells with 0 read counts. Methods evaluated: original scRNA-seq data; CellDEEP (k-means or random selection, sum or mean aggregation, 10 cells per metacell); and pseudobulk.

To better understand these differences, we examined the negative control comparisons in two clusters in greater detail by visualizing the *P*-value distributions (Supplementary Figs S6 and S7). In an ideal negative control experiment, only 5% of *P*-values should fall below 0.05, and their distribution should be uniform between 0 and 1. In the CD14^+^ monocyte cluster from the COVID-19 dataset, single-cell MAST again performed poorly, identifying 8129 false positives in the negative control comparison (Supplementary Fig. S6). While CellDEEP (R-sum) only partially mitigated this issue, reducing false positives by ~10%, a different CellDEEP configuration emerged as a top performer. Across all real datasets and cell types tested, CellDEEP (R-mean, DESeq2) consistently achieved the lowest FPR. Its performance was superior to all scRNA-seq-based methods and comparable to standard pseudobulk methods in controlling false positives (Fig. 3A). These results were mirrored in the RA TREM2^high^ cluster.

Furthermore, it was unexpected that the R-mean (CellDEEP) controlled FP better than the R-sum (CellDEEP), unlike in the simulation datasets. Our hypothesis was that in real single cell data, ‘noise’ was higher when summing the counts to form metacells and was averaged out in the mean. Hence, we compared the dropout rate of the cells for the different methods in the CD14^+^ and TREM2^high^ datasets (Fig. 3B). As expected, the scRNA-seq shows high, and the pseudobulk low, dropout rates. CellDEEP with k-means selection and sum aggregation (K-sum) behaves similarly to pseudobulk, although the median is higher compared to pseudobulk. In contrast, the values of CellDEEP with k-means selection and mean aggregation read count (K-mean) approach 1.

This effect is caused by rounding during averaging and raw count generation. When the overall read count is very low (e.g. a mean of 0.3), rounding converts the value to 0. As a result, some lowly expressed genes appear to lose signal. However, such genes with low counts are often dominated by technical noise rather than true biological signal [25]. Therefore, rounding primarily removes background noise instead of meaningful expression. In the simulations (Supplementary Fig. S5), we can see the similar trend. However, the Muscat dropout rates have less spread, and the Zimmerman values are similar to the real dropout rates.

### True positive

As the biological processes underlying RA and COVID-19 are well characterized, established immune pathways provide a qualitative benchmark for expected DE patterns. To assess our ability to detect biologically meaningful signal, we examined differentially expressed genes (DEGs) within canonical immune pathways known to be relevant to each condition using two complementary metrics.

First, we calculated PRR (Fig. 3A and Supplementary Fig. S8) as the proportion of expected pathways that each method successfully detected from our curated list of true positive GO terms. This metric reveals how comprehensively a method captures known biology. Here, pseudobulk methods showed a critical limitation: they have reduced sensitivity, as shown by others, recovering substantially fewer expected DEG/GO than single-cell methods or CellDEEP.

The only cluster that performs similarly is the NK cells, which has a very limited number of cells (Supplementary Table S1). The traditional single cell methods are generally close to the CellDEEP results, ranging from 0.5 to 0.7 in most cases, however, as noted before, many false positive results were observed for scRNAseq-based methods.

Second, we calculated signal density (Fig. 4) as the proportion of each methods called DE genes that fell within expected pathways. This metric distinguishes methods that genuinely detect relevant biology from those that simply call more genes by chance. We explored the CD14^+^ monocytes in more detail, where pseudobulk-based methods recovered roughly half the number of true positive pathway-associated compared to single-cell methods or CellDEEP (Fig. 4A). While all methods identified core pathways such as ‘defense response to virus’, scRNA-seq and CellDEEP methods additionally recovered a broader set of immune processes involved in the antiviral response. These included additional immune processes central to antiviral defence (‘regulation of innate immune response’, ‘positive regulation of cytokine production involved in inflammatory response’) as well as key effector mechanisms (‘antigen processing and presentation via MHC class I’, ‘NF-kappaB binding’) (Supplementary Table S4). A similar pattern was observed for TREM2^high^ macrophages. Notably, pseudobulk DESeq2 showed an artificially high signal density in this cluster (Fig. 4B), which was driven by its extremely conservative gene calls, as reflected in its low PRR for the same cluster (Fig. 3A).

**Figure 4.**
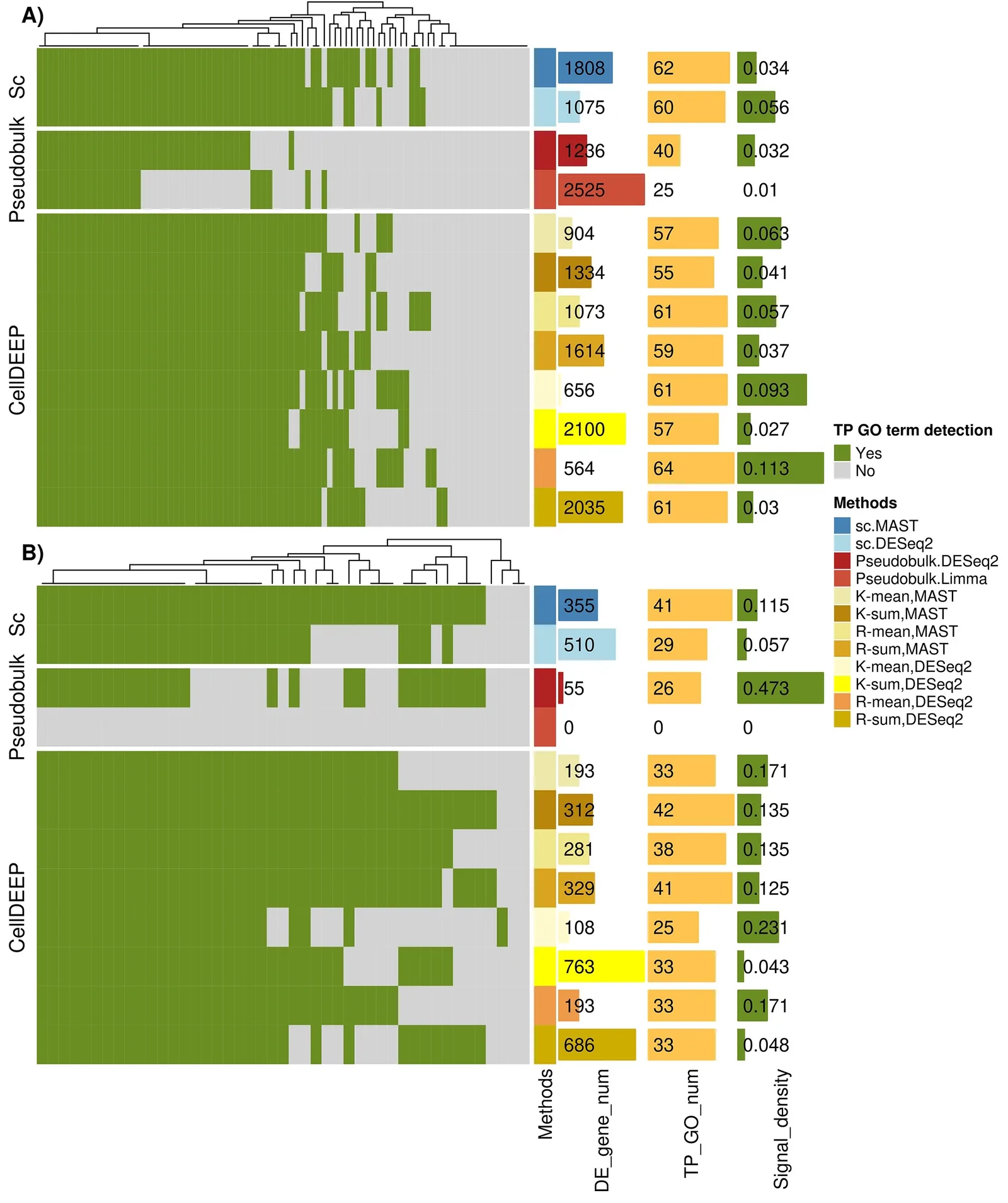
Benchmarking DE methods by true positive GO term detection. (**A**) COVID19 CD14^+^ monocytes and (**B**) RA TREM2^high^ Macrophage. For each method, bars include: total number of DE genes called (DE_gene_num, coloured by method); number of true positive (TP_GO_num) GO terms detected (light yellow); and signal density (Signal_density, green), calculated as the number of TP GO terms detected divided by total DE gene count. Methods compared: scRNA-seq (MAST, DESeq2), pseudobulk (DESeq2, limma), and CellDEEP with various parameter combinations (selection: random or k-means; aggregation: sum or mean; downstream DE tool: DESeq2 or limma; 10 cells per metacell).

Across all real datasets, CellDEEP methods were among the top performers by both metrics. However, the sum aggregation methods consistently generated more false positives (Supplementary Fig. S8), suggesting that mean aggregation provides better specificity in real data.

Overall, CellDEEP achieved the best balance between true positive detection and false positive control. By aggregating cells into replicated metacells, it preserves sufficient biological heterogeneity to match the sensitivity of single-cell methods while stabilizing expression estimates to approach the specificity of pseudobulk approaches.

## Discussion

Although single-cell transcriptomics has substantially advanced our understanding of cellular function, the robust detection of DEGs remains an open challenge. Previous studies have reported that single-cell DE methods tend to overpredict DEGs, whereas pseudobulk approaches, while better controlling false positives, may suffer from reduced sensitivity [3]. Several reports have suggested that pseudobulk represents a preferable strategy due to improved control of false discovery rates. However, these conclusions have largely been based on simulated data and benchmark where pseudobulk has been treated as gold standard, as establishing a reliable ground truth set in real scRNA-seq datasets remains inherently difficult.

First, we developed CellDEEP, an approach designed to combine the strengths of single-cell and pseudobulk frameworks. To address the limitation of real data truth sets, we introduce an alternative strategy for performance assessment that leverages real datasets to approximate ground truth.

Our results demonstrate that no single method consistently outperforms all others. Conventional single-cell methods identify a large number of candidate genes but exhibit elevated FPR, whereas pseudobulk approaches control false positives better, at the cost of reduced sensitivity. We acknowledge that the performance of existing methods could potentially be improved through more extensive parameter tuning, for example, incorporating latent variables in MAST or including metadata covariates in pseudobulk models. However, such optimizations are equally applicable to CellDEEP and do not alter the relative performance observed in our benchmarks.

Importantly, our tool achieves a more balanced trade-off between these two extremes. Across two simulation frameworks, pseudobulk approaches showed higher overall accuracy but lower sensitivity than CellDEEP. As illustrated in Fig. 1C and D, excessive pooling of cells reduces statistical power across methods. While the precise mechanistic explanation remains unclear, it is evident that simulation frameworks do not fully capture the complexity and variability inherent to real biological data [26, 27]. This raises a fundamental question: how can a meaningful truth set be established using real datasets?

To address this, we propose two complementary strategies for approximating ground truth. First, we analysed large single-cell datasets from COVID-19 and RA by comparing samples within the same condition, thereby generating two biologically equivalent groups. In this setting, no true DE exists, as the comparison is constructed under the null hypothesis. Examination of *P*-value distributions (Supplementary Figs S6 and S7) revealed that single-cell methods produced a substantial excess of low *P*-values, consistent with inflated false positives. Pseudobulk methods displayed a more uniform *P*-value distribution, whereas CellDEEP exhibited intermediate behaviour.

To estimate true positive rates, we adopted a biologically informed strategy. For each disease, we identified Gene Ontology terms known to be relevant to disease pathology and defined genes within these categories as true positives. Under this framework, CellDEEP demonstrated strong performance, comparable to single-cell methods. Consistent with previous reports, pseudobulk approaches showed reduced sensitivity; however, this reduction was more pronounced in real datasets than in simulations.

Although CellDEEP performs robustly overall, performance varies across datasets and parameter choices. Importantly, the optimal aggregation strategy is context-dependent: sum aggregation consistently outperformed mean in simulations, whereas in real datasets mean aggregation with DESeq2 achieved the lowest FPR. This discrepancy likely reflects the higher noise levels in real data, which averaging may help to mitigate. We did not observe a consistent pattern indicating that a specific CellDEEP implementation was universally optimal. For example, it remains unclear whether aggregation strategies such as R-sum or R-mean perform better under specific data-generating scenarios. A key point is that CellDEEP’s advantage over pseudobulk lies not in superior null calibration, but in improved sensitivity. Pseudobulk methods recover substantially fewer true positive GO terms than CellDEEP, indicating that their specificity comes at the cost of reduced sensitivity. CellDEEP offers a middle ground: it maintains good false positive control while recovering more biologically relevant signal. Therefore, rather than advocating for a single superior method, we recommend the complementary use of multiple approaches, with careful consideration of their respective strengths and limitations. The principal advantage of CellDEEP lies in its balanced control of false positives and true positives.

In conclusion, we present CellDEEP, a novel framework for DE analysis that achieves a favourable balance between sensitivity and specificity. Importantly, our work also introduces an alternative evaluation strategy based on real datasets rather than simulations alone, which often fail to capture the full complexity of biological variability. We suggest that CellDEEP be used alongside established methods to assess the robustness of findings and to better characterize false and true positive rates in single-cell transcriptomic analyses.

## Supplementary Material

lqag128_Supplemental_Files

## Data Availability

The CellDEEP package is freely available from CRAN (https://cran.r-project.org/web/packages/CellDEEP) and the source code is hosted on GitHub (https://github.com/sii-scRNA-Seq/CellDEEP). The specific version used in this study is archived on Zenodo (https://zenodo.org/records/22028542). Code to reproduce all analyses presented in this paper is available at Zenodo (https://zenodo.org/records/22028483). Simulated data used during analysis are also available on Zenodo (https://zenodo.org/records/18863779).
