# Supplementary material for: Cell DiffErential Expression by Pooling (CellDEEP) highlights issues in differential gene expression in scRNA-seq
Source: NAR Genom Bioinform. 2026 Oct 21;8(4):lqag128. doi: 10.1093/nargab/lqag128 (PMC13684280; doi:10.1093/nargab/lqag128)
Supplement: Supplementary file 1 [file lqag128_supplemental_files.zip › CellDEEP suppl table 1-2-3-5 130726.docx]

| **Cell type** | **Group** | **Replicant count** | **Total cells** | **Mean cells per replicate** | **SD cells per replicate** |
| --- | --- | --- | --- | --- | --- |
| COVID-19 B cell | Healthy | 24 | 3727 | 155.29 | 161.48 |
|  | Severe | 7 | 2516 | 359.43 | 302.41 |
| COVID-19 T cells CD8^+^ | Healthy | 21 | 9385 | 446.90 | 328.35 |
|  | Severe | 7 | 3013 | 430.43 | 503.67 |
| COVID-19 NK CD56^high^ cells | Healthy | 24 | 1099 | 45.79 | 37.44 |
|  | Severe | 7 | 342 | 48.86 | 26.34 |
| COVID-19 monocytes CD14^+^ | Healthy | 24 | 5162 | 215.08 | 250.09 |
|  | Severe | 7 | 3706 | 529.43 | 514.66 |

***Supplementary Table 1.*** **COVID-19 dataset summary**. The replicate number and statistical summaries of cell distribution in the COVID-19 PBMC data.

| **Cell Type** | **Group** | **Replicates Number** | **Total Cells** | **Mean Cells Per Replicate** | **SD Cells Per Replicate** |
| --- | --- | --- | --- | --- | --- |
| TREM2^high^ | Healthy | 4 | 1775 | 443.75 | 229.54 |
|  | Active RA | 6 | 860 | 143.33 | 78.44 |
| HLA^high^ | Healthy | 4 | 893 | 223.25 | 138.33 |
|  | Active RA | 6 | 1437 | 239.5 | 96.55 |

***Supplementary Table 2****.* **Rheumatoid arthritis dataset summary**. The replicate number and summary statistics for cell-distribution in the RA data.

| **Cluster** | **Group** | **Samples** |
| --- | --- | --- |
| B cell | 1 | MH8919333, MH8919332, MH8919227, MH8919179, MH8919177 |
|  | 2 | MH8919283, MH8919178, MH8919226, MH8919282, MH8919176 |
| CD8^+^ T cells | 1 | MH9179822, BGCV12_CV0178, BGCV08_CV0915, MH8919179, MH8919332 |
|  | 2 | BGCV06_CV0178, newcastle65, BGCV14_CV0940, BGCV10_CV0939, BGCV05_CV0929 |
| NK cells CD56^high^ | 1 | MH8919333, MH8919332, MH8919227, MH8919179, MH8919177 |
|  | 2 | MH8919283, MH8919178, MH8919226, MH8919282, MH8919176 |
| CD14^+^ Monocytes | 1 | MH8919333, MH8919332, MH8919227, MH8919179, MH8919177 |
|  | 2 | MH8919283, MH8919178, MH8919226, MH8919282, MH8919176 |
| Macrophage TREM2^high^ | 1 | SA144, SA145, SA222 |
|  | 2 | SA151, SA166, SA227 |
| Macrophage HLA^high^CLEC10A^high^ | 1 | SA144, SA145, SA222 |
|  | 2 | SA151, SA166, SA227 |

***Supplementary Table 3****.* **Null hypothesis tests summary**. The table lists the sample IDs used for the p-value null hypothesis tests. For each cluster, samples from the same biological group were randomly assigned to Group 1 and Group 2, with 5 samples in each group for the COVID-19 dataset and 3 samples in each group for the RA dataset, to construct the null hypothesis test dataset.

| **Simulation framework** | **Aggregation parameter** | **Cell selection parameter** | **Accuracy** | |
| --- | --- | --- | --- | --- |
|  |  |  | **MAST** | **DESeq2** |
| Muscat | mean | k-means | 0.95 | 0.96 |
|  |  | Random | 0.95 | 0.96 |
|  | Sum | k-means | 0.96 | **0.99** |
|  |  | Random | 0.97 | **0.99** |

***Supplementary table 5.* Comparison of CellDEEP strategies for accuracy in Muscat simulation.** Read-count aggregation methods (Mean vs. Sum) and cell-selection approaches (K-means vs. Random) are evaluated, result split by DE methods (MAST vs DESeq2). Values represent the median accuracy. 10 cells were aggregated.
