# Supplementary material for: Cell DiffErential Expression by Pooling (CellDEEP) highlights issues in differential gene expression in scRNA-seq
Source: NAR Genom Bioinform. 2026 Oct 21;8(4):lqag128. doi: 10.1093/nargab/lqag128 (PMC13684280; doi:10.1093/nargab/lqag128)
Supplement: Supplementary file 1 [file lqag128_supplemental_files.zip › CellDEEP_Figure_panel_July_v3.pdf]

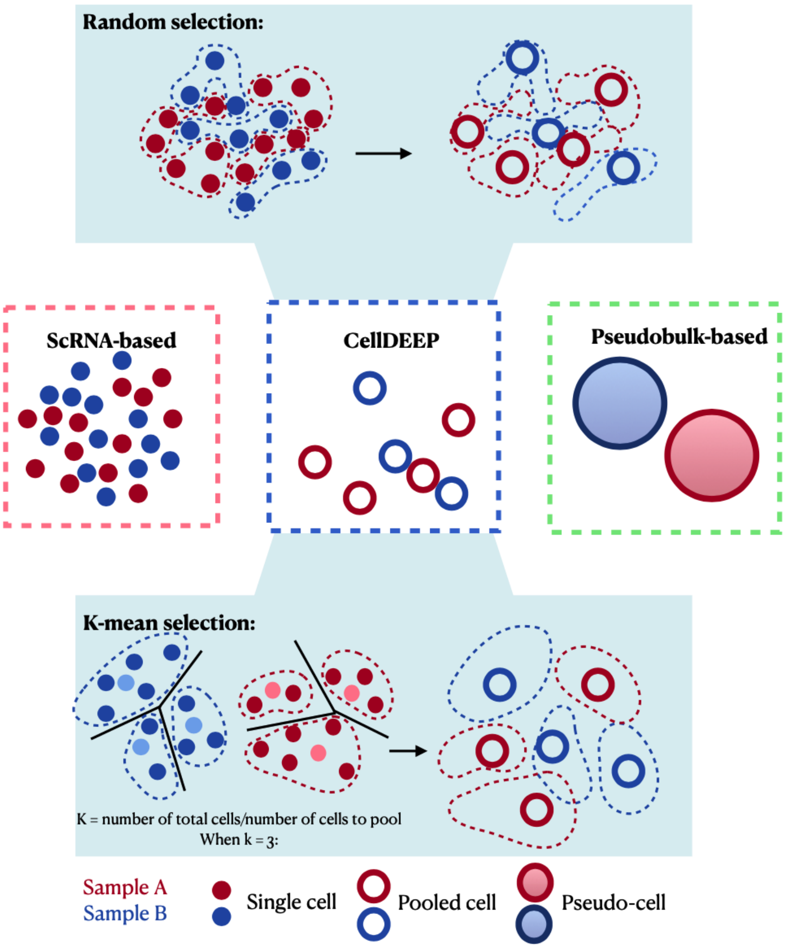

Supplementary Figure 1. Overview of CellDEEP. Unlike scRNA-seq (which analyses individual cells) or pseudobulk approaches (which aggregate all cells in a cluster), CellDEEP aggregates individual cells into metacells using two alternative strategies: random pooling (top) or k-means clustering (bottom).

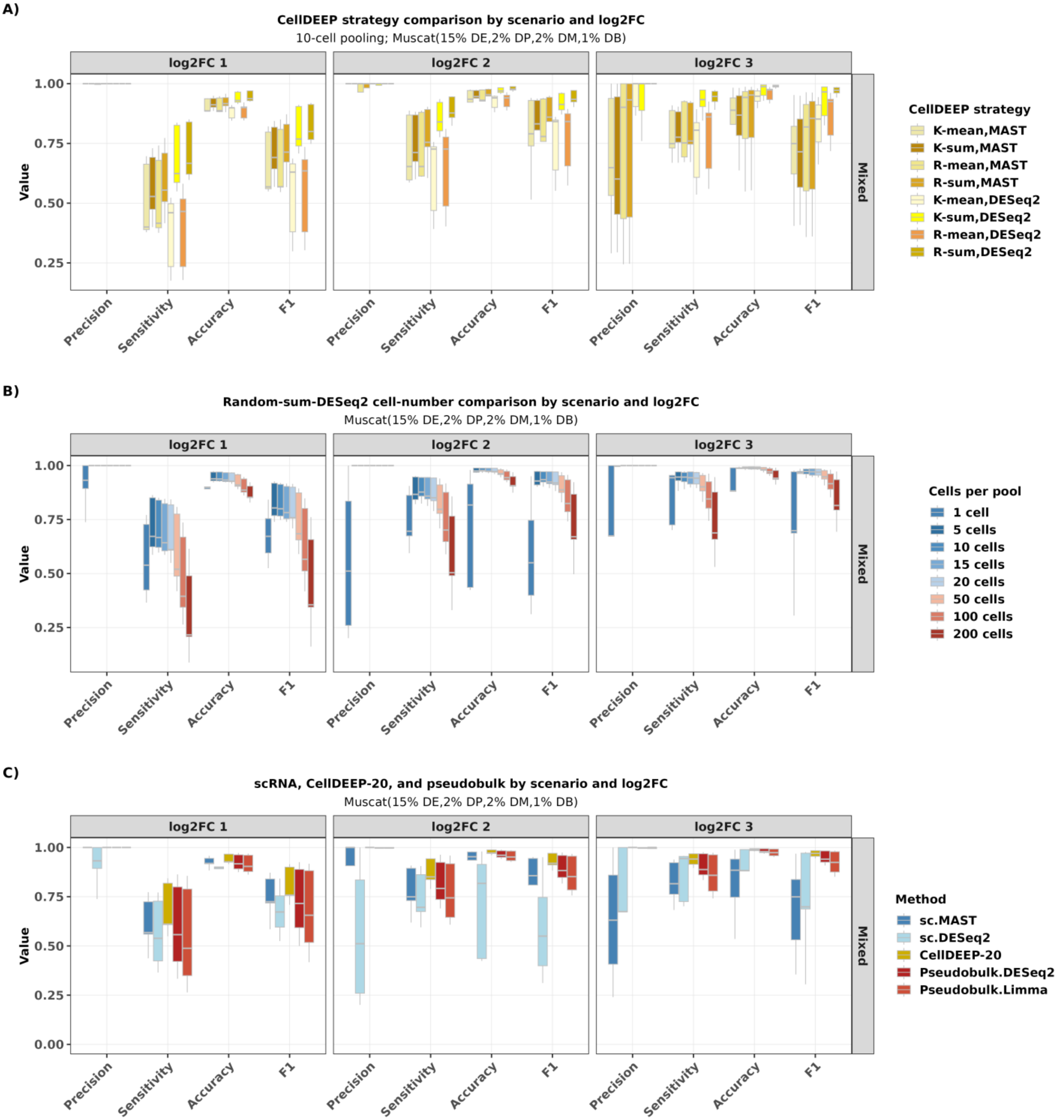

Supplementary Figure 2. **Benchmarking CellDEEP against scRNA and pseudobulk methods in mix-weighted simulated data.** Based on the Muscat mix-weighted simulation (15% DE, 2% DP, 2% DM, 1% DB). Performance was evaluated using precision, sensitivity, accuracy, and F1-score. **A)** Performance of CellDEEP under different cell-selection and aggregation strategies. K- denotes k-means-based cell selection, R- denotes random cell selection, and sum and mean denote count aggregation by summing or averaging, respectively. **B)** Effect of metacell size on CellDEEP performance using random cell selection, summed-count aggregation, and DESeq2. Metacell sizes of 1, 5, 10, 15, 20, 50, 100, and 200 cells were tested. **C)** Comparison of CellDEEP(Random-Sum-DESeq2, 20 cells), scRNA-based methods(MAST&DESeq2), and pseudobulk-based methods (DESeq2&Limma).

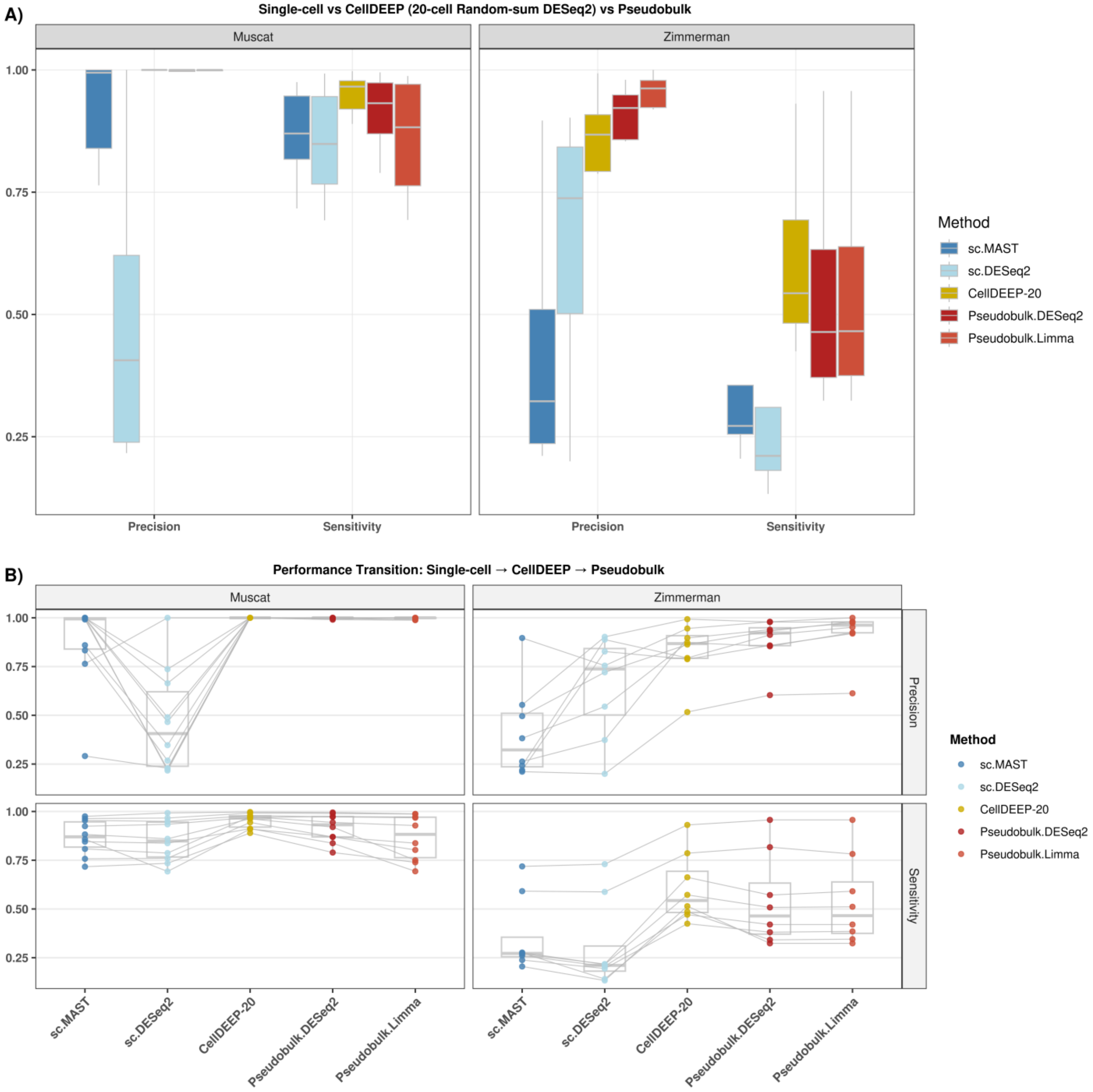

Supplementary Figure 3. **Benchmarking CellDEEP against scRNA and pseudobulk methods in DE only simulated data. A)** Precision and sensitivity across Muscat and Zimmerman simulation frameworks for CellDEEP (random selection, sum aggregation, 20 cells per metacell, DESeq2), scRNA-seq (DESeq2), and pseudobulk (DESeq2). **B)** Precision and sensitivity across the same datasets connected by lines, comparing CellDEEP (Random–Sum–DESeq2), scRNA-seq (DESeq2), and pseudobulk (DESeq2) in both simulation frameworks. Each line represents a shared dataset.

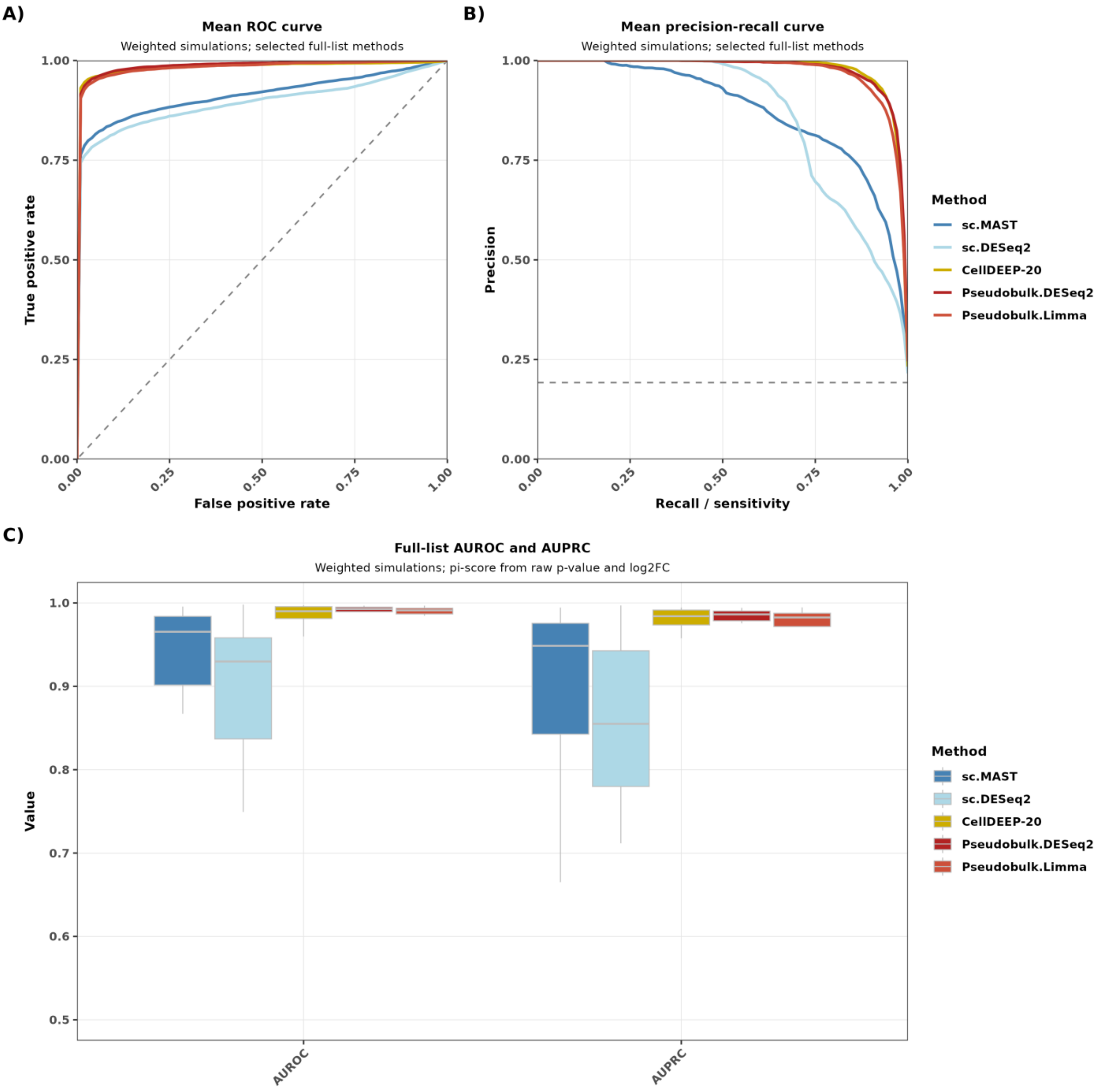

Supplementary Figure 4. **Benchmarking CellDEEP against scRNA and pseudobulk methods by ROC/PRC in mix-weighted simulated data.** Based on the Muscat mix-weighted simulation (15% DE, 2% DP, 2% DM, 1% DB). Comparison of CellDEEP (Random-Sum-DESeq2, 20 cells), scRNA-based methods (MAST and DESeq2), and pseudobulk-based methods (DESeq2 and limma). **A)** Mean ROC curves. **B)** Mean PRC. **C)** AUROC and AUPRC across simulations.

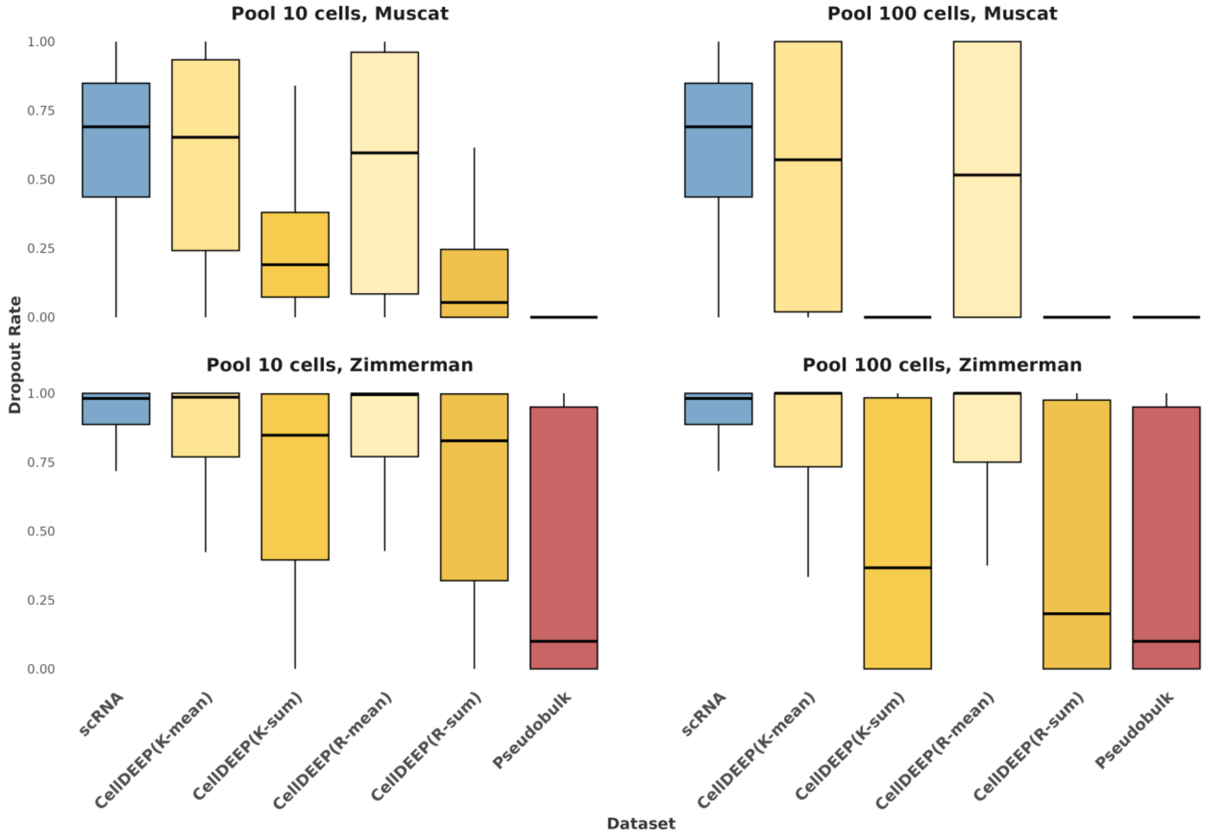

Supplementary Figure 5. Gene dropout comparison across simulated datasets. *For each gene, dropout rate was calculated as the proportion of cells with 0 read count.* Results are shown separately for Zimmerman and Muscat simulation frameworks and for metacell sizes of 10 and 100 cells. Methods compared: original scRNA-seq data; CellDEEP (k-means or random selection, sum or mean aggregation, 10 or 100 cells per metacell); and pseudobulk.

COVID-19 CD14⁺ Monocytes

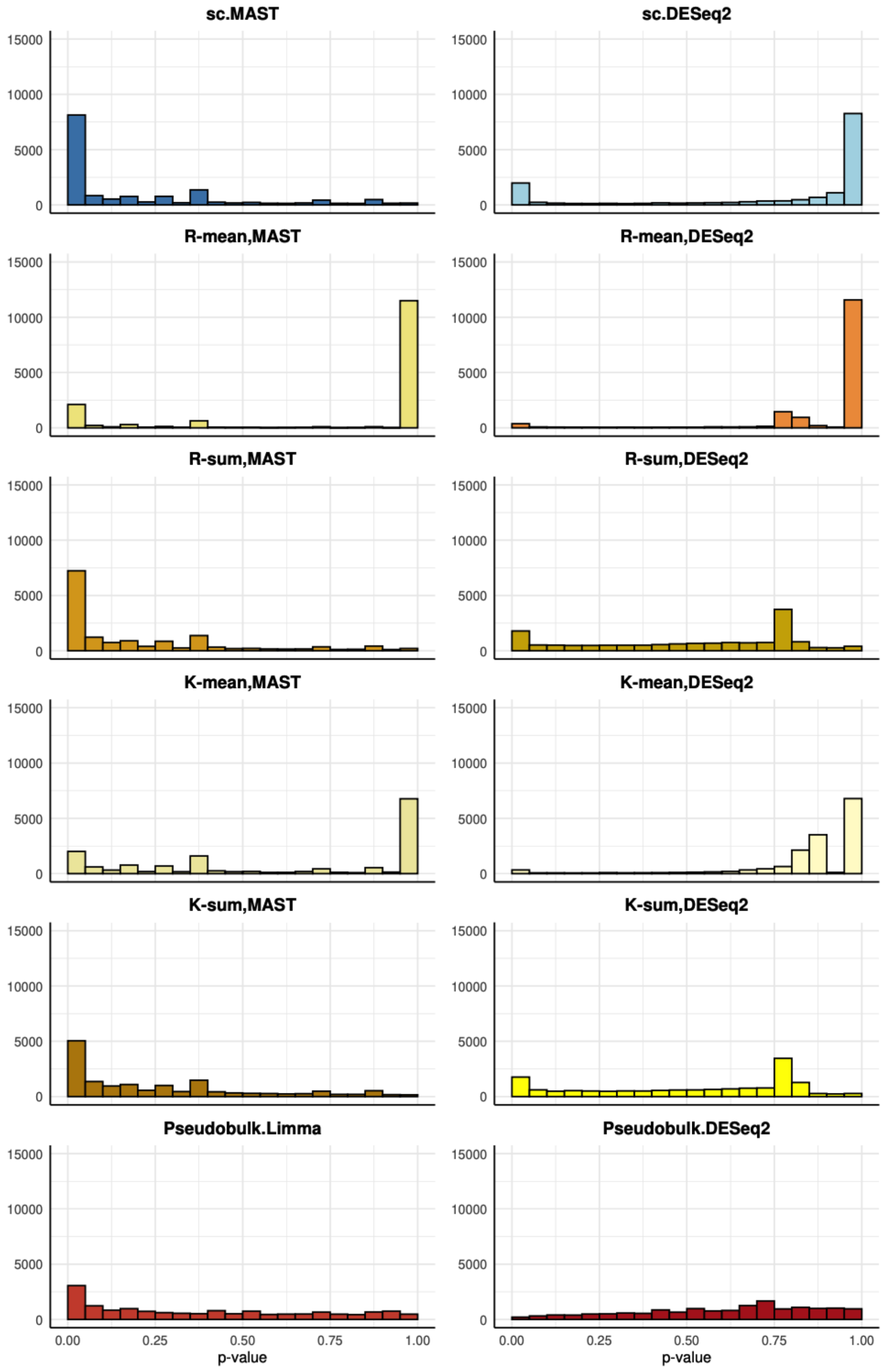

Supplementary Figure 6.* P-value distributions under null hypothesis comparisons in COVID-19 CD14⁺ *Monocytes.
Histograms show p-values from negative control comparisons (same-condition replicate splits) for each DE method. An ideal null distribution should be uniform with no enrichment of low p-values. Methods evaluated: scRNA-seq (MAST, DESeq2); pseudobulk (DESeq2, Limma); CellDEEP (random or k-means selection, sum or mean aggregation, 10 cells per metacell) with DESeq2 or MAST.

RA Macrophage TREM2^high cell

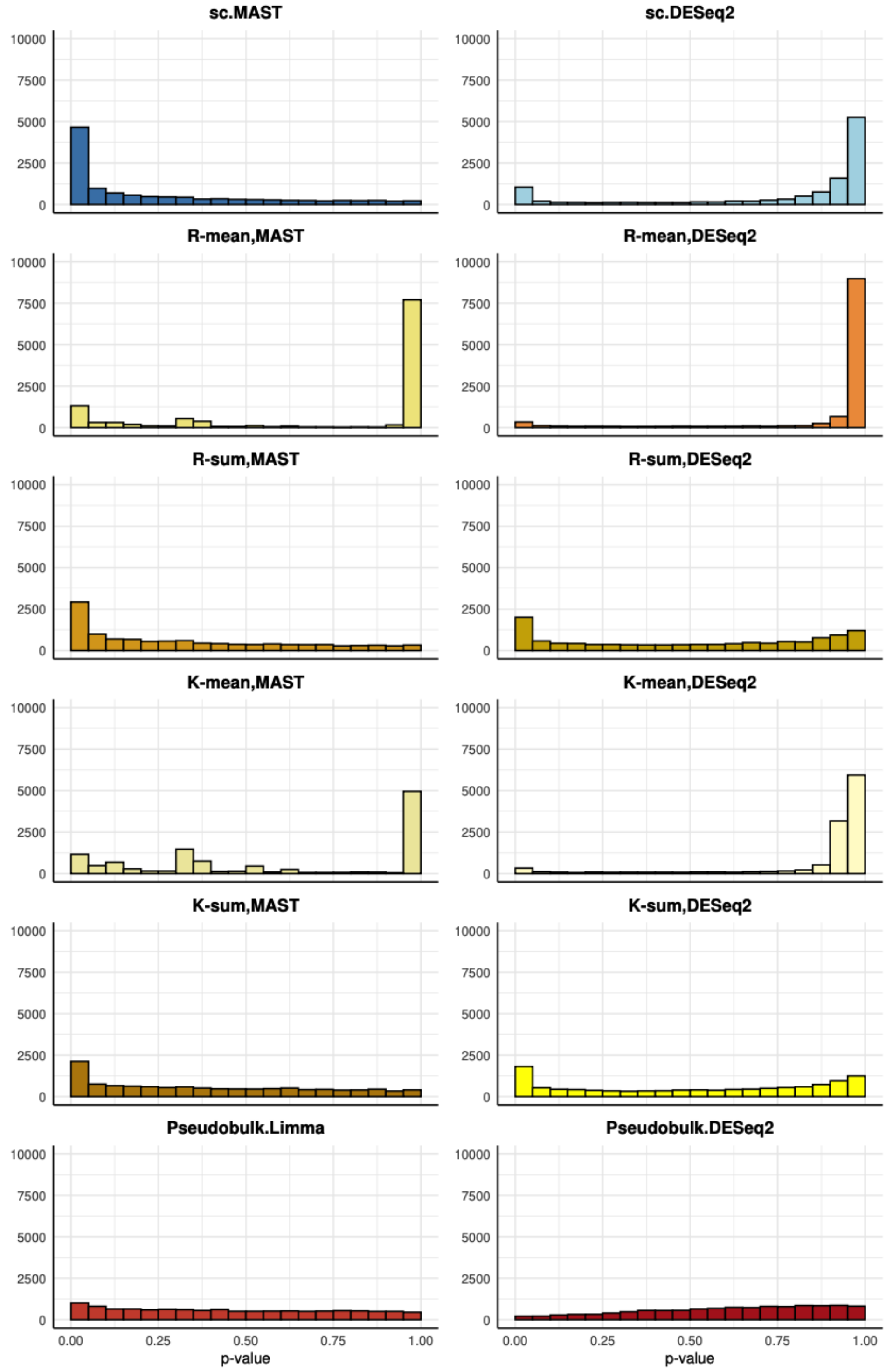

Supplementary Figure 7. **P-value distributions under null hypothesis comparisons in RA TREM2^{high} macrophages.** Histograms show p-values from negative control comparisons (same-condition replicate splits) for each DE method. An ideal null distribution should be uniform with no enrichment of low p-values. Methods evaluated: scRNA-seq (MAST, DESeq2); pseudobulk (DESeq2, Limma); CellDEEP (random or k-means selection, sum or mean aggregation, 10 cells per metacell) with DESeq2 or MAST.

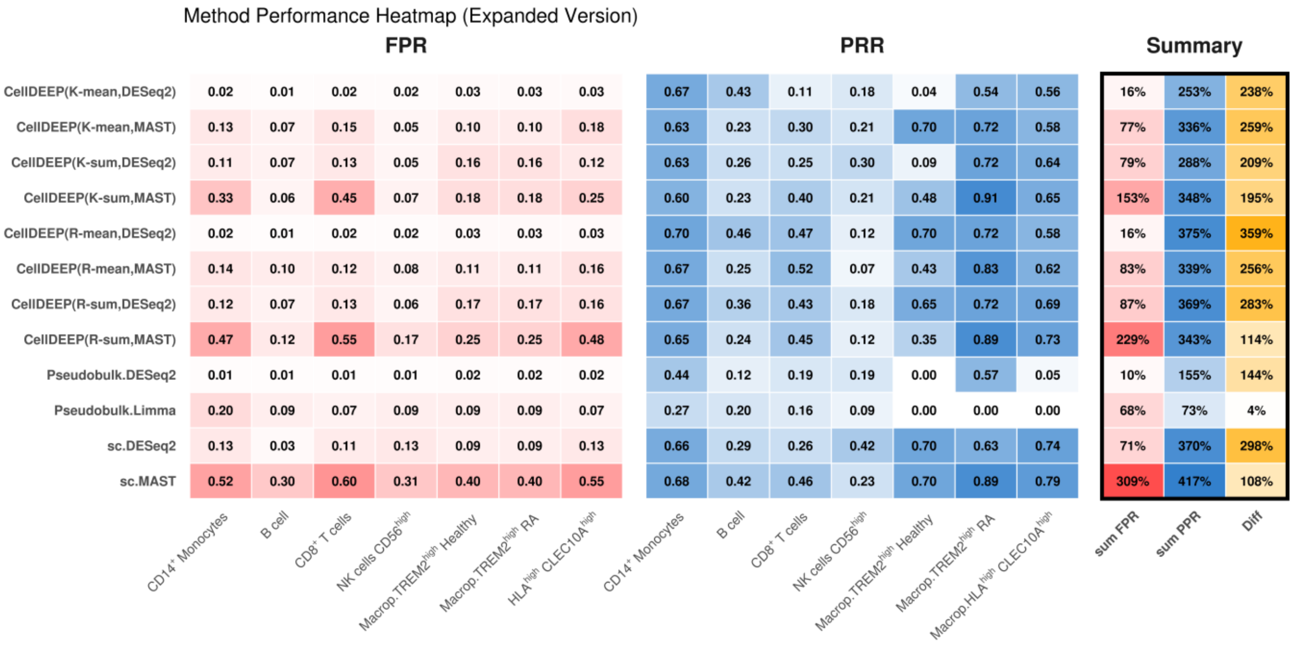

Supplementary Figure 8. **Full method evaluation on published datasets: false positive rate and pathway recovery rate.** For each method, we calculated false positive rate (FPR) from negative control comparisons and pathway recovery rate (PRR) from GO enrichment analysis. Sum FPR and Sum PRR represent totals across all datasets (percentages). Diff (Sum PRR − Sum FPR) indicates the net balance between true positive detection and false positive control. Methods evaluated: scRNA-seq (MAST, DESeq2); pseudobulk (DESeq2, Limma); CellDEEP (random or k-means selection, sum or mean aggregation, 10 cells per metacell) with DESeq2 or Limma.
