# Supplementary material for: Cell DiffErential Expression by Pooling (CellDEEP) highlights issues in differential gene expression in scRNA-seq
Source: NAR Genom Bioinform. 2026 Oct 21;8(4):lqag128. doi: 10.1093/nargab/lqag128 (PMC13684280; doi:10.1093/nargab/lqag128)
Supplement: Supplementary file 1 [file lqag128_supplemental_files.zip › CellDEEP_suppl table 6-7.docx]

| Simulation framework | Reference dataset | Biological comparison represented by reference | Number of groups | Replicates per group | Cells | Genes | Signal proportions | Effect size setting | Datasets generated |
| --- | --- | --- | --- | --- | --- | --- | --- | --- | --- |
| Muscat | muscat_original | PBMC from lupus patients before and after 6h IFN-β treatment | 2 | 10 | 4000 | 2000 | 20% DE, 0% DP, 0% DM, 0% DB, 40% EE, 40% EP | log2FC = 2 and 3 | 2 |
|  | alpha_cells | Pancreatic alpha cells from Zimmerman reference | 2 | 10 | 4000 | 2000 | 20% DE, 0% DP, 0% DM, 0% DB, 40% EE, 40% EP | log2FC = 2 and 3 | 2 |
|  | covidCD14 | CD14+ monocytes from healthy and severe COVID-19 patients | 2 | 10 | 4000 | 2000 | 20% DE, 0% DP, 0% DM, 0% DB, 40% EE, 40% EP | log2FC = 2 and 3 | 2 |
|  | covidCD8 | CD8+ T cells from healthy and severe COVID-19 patients | 2 | 10 | 4000 | 2000 | 20% DE, 0% DP, 0% DM, 0% DB, 40% EE, 40% EP | log2FC = 2 and 3 | 2 |
|  | macrophage | TREM2low macrophage cluster from active rheumatoid arthritis patients and healthy donors | 2 | 10 | 4000 | 2000 | 20% DE, 0% DP, 0% DM, 0% DB, 40% EE, 40% EP | log2FC = 2 and 3 | 2 |
| Zimmerman | alpha_cells | Pancreatic alpha cells from Zimmerman reference | 2 | 10 | 4000 | 2000 | 20% DE | FC = 4 and 8 | 2 |
|  | covidCD14 | CD14+ monocytes from healthy and severe COVID-19 patients | 2 | 10 | 4000 | 2000 | 20% DE | FC = 4 and 8 | 2 |
|  | covidCD8 | CD8+ T cells from healthy and severe COVID-19 patients | 2 | 10 | 4000 | 2000 | 20% DE | FC = 4 and 8 | 2 |
|  | macrophage | TREM2low macrophage cluster from active rheumatoid arthritis patients and healthy donors | 2 | 10 | 4000 | 2000 | 20% DE | FC = 4 and 8 | 2 |

**Supplementary Table 6. Summary of all simulation setting in pure DE test.**

Summary of pure differential-expression simulations generated with the Muscat and Zimmerman frameworks. All datasets contained two groups, 10 replicates per group, 4,000 cells, 2,000 genes, and 20% differentially expressed genes, with two effect-size settings per reference dataset.

| Simulation framework | Reference dataset | Biological comparison represented by reference | Group number | Replicates per group | Cells | Genes | Signal proportions | logFC setting | Datasets generated |
| --- | --- | --- | --- | --- | --- | --- | --- | --- | --- |
| Muscat | Muscat original | PBMC from lupus patients before and after 6h IFN-β treatment | 2 | 10 | 4000 | 2000 | 15% DE, 2% DP, 2% DM, 1% DB, 40% EE, 40% EP | log2FC = 1, 2, and 3 | 3 |
| Muscat | alpha_cells | Pancreatic alpha cells from Zimmerman reference | 2 | 10 | 4000 | 2000 | 15% DE, 2% DP, 2% DM, 1% DB, 40% EE, 40% EP | log2FC = 1, 2, and 3 | 3 |
| Muscat | covidCD14 | CD14+ monocytes from healthy and severe COVID-19 patients | 2 | 10 | 4000 | 2000 | 15% DE, 2% DP, 2% DM, 1% DB, 40% EE, 40% EP | log2FC = 1, 2, and 3 | 3 |
| Muscat | covidCD8 | CD8+ T cells from healthy and severe COVID-19 patients | 2 | 10 | 4000 | 2000 | 15% DE, 2% DP, 2% DM, 1% DB, 40% EE, 40% EP | log2FC = 1, 2, and 3 | 3 |
| Muscat | macrophage | TREM2low macrophage cluster from active rheumatoid arthritis patients and healthy donors | 2 | 10 | 4000 | 2000 | 15% DE, 2% DP, 2% DM, 1% DB, 40% EE, 40% EP | log2FC = 1, 2, and 3 | 3 |

**Supplementary Table 7. Summary of all simulation setting in mix-weighted test.**

Overview of Muscat mix-weighted simulation datasets generated from five reference datasets. Each simulation included two groups, 10 replicates per group, 4,000 cells, and 2,000 genes, with predefined signal proportions and log₂ fold changes of 1, 2, or 3.
